# IgM monoclonal gammopathy with heavy-and-light-chain amyloidosis resembling fibrillary glomerulonephritis determined by tandem mass spectrometry: a case report

**DOI:** 10.1186/s12882-020-01851-4

**Published:** 2020-05-24

**Authors:** Misa Adachi, Mineaki Kitamura, Kumiko Muta, Akihiro Maekawa, Tadashi Uramatsu, Masato Tadokoro, Satoshi Funakoshi, Satoshi Hisano, Naomi Kuwahara, Akira Shimizu, Hiroshi Mukae, Tomoya Nishino

**Affiliations:** 1 Department of Nephrology, Nagasaki University Graduate School of Biomedical Sciences, Nagasaki, Japan; 2 Department of Nephrology, Nagasaki Renal Center, Nagasaki, Japan; 3 Department of Nephrology, Nagasaki Medical Center, Omura, Japan; 4 Department of Nephrology, Koritsu Shin-Obama Hospital, Nagasaki, Japan; 5 Department of Pathology, University of Occupational and Environmental Health School of Medicine, Kitakyushu, Japan; 6 Department of Analytic Human Pathology, Nippon Medical School, Tokyo, Japan; 7 Department of Respiratory Medicine, Nagasaki University Graduate School of Biomedical Sciences, Nagasaki, Japan

**Keywords:** Fibrillary glomerulonephritis, IgM gammopathy, Immunoglobulin heavy-and-light-chain amyloidosis, Laser micro dissection, Mass spectrometry

## Abstract

**Background:**

Fibrillary glomerulonephritis (FGN) is distinguished from amyloidosis by thicker fibrils and the lack of staining with histochemical dyes typically reactive with amyloid. However, congophilic FGN has been proposed recently and adding laser microdissection followed by mass spectrometry (LMD/MS) to conventional pathological methods would be helpful to diagnose FGN. Here, we report a patient initially diagnosed with FGN whose final pathological diagnosis was changed to immunoglobulin heavy-and-light-chain amyloidosis (AHL) after LMD/MS.

**Case presentation:**

A 75-year-old male developed nephrotic syndrome. Protein electrophoresis showed IgM κ type M proteinemia and he was diagnosed with IgM monoclonal gammopathy. A renal biopsy was performed and pathological examination showed marked periodic acid-Schiff-positive enlargement of the mesangial region and silver stain positivity, but weak direct fast scarlet staining. Immunofluorescence analysis showed monoclonal deposition of IgM-κ chain in the glomerulus. Under electron microscopy, the fibrils were about 20 nm in diameter, which was thicker than typical amyloid fibrils. Based on these findings, the patient was diagnosed with FGN. Although cyclophosphamide and prednisolone were administered, his renal function deteriorated and progressed to end stage renal disease requiring maintenance hemodialysis. As congophilic FGN has been recognized since 2018, Congo red staining and LMD/MS were performed. The Congo red staining was positive and LMD/MS results indicated that this was a case of AHL.

**Conclusions:**

We reported a case of μ and κ chain AHL resembling FGN requiring LMD/MS for definitive diagnosis. Since FGN and amyloidosis exhibit pathological findings, even if Congo red staining is positive, LMD/MS needs to be considered in cases atypical pathological findings, such as silver stain positivity or thicker fibrils.

## Background

Amyloidosis is a clinical disorder that causes organ damage through the extracellular deposition of amyloid, in which soluble proteins become insoluble aggregates with a β-pleated structure. Amyloid fibrils are pathologically characterized as non-branching fibrils that are 7.5 to 10 nm in diameter on electron microscopy, and exhibit a blue-green birefringence after Congo red staining [1, 2]. In contrast, fibrillary glomerulonephritis (FGN) is characterized by the deposition of fine fibers on the glomeruli that have a diameter of 15 to 25 nm. They are found in approximately 1% of all adult renal biopsies and while their pathological features are similar to amyloidosis, the randomly arranged fibrils are thicker and they are not stained by histochemical dyes [3, 4].

New insights into FGN have been recently reported, including the specificity of the DnaJ homolog subfamily B member 9 (DNAJB9) in FGN [5] and congophilic FGN [6]. According to case series on congophilic FGN [6], laser microdissection followed by mass spectrometry (LMD/MS) is required to distinguish it from renal amyloidosis. Nasr et al. reported that LMD/MS is also useful for the diagnosis and classification of immunoglobulin heavy-and-light chain-amyloidosis (AHL) [7]. AHL is a rare renal disease that can be difficult to diagnose using conventional pathological methods [7].

Here, we report a patient that was initially diagnosed with FGN based on scarcely positive in direct fast scarlet (DFS) staining (also known as Dylon staining, which can be a substitute for Congo red staining [8]) visualized by polarizing microscopy, and the presence of fibrils thicker than typical amyloid fibrils on electron microscopy. However, LMD/MS analysis led to a change in the final diagnosis to AHL.

## Case presentation

A 75-year-old man visited our hospital because of exacerbation of lower extremity edema that he had noticed 3 months before admission. He had received annual medical checks and had no specific medical history. His body weight had increased by 10 kg over the past 5 months, and his systemic edema was remarkable. Urinary protein was 9.9 g/day, no microscopic hematuria was detected, and his serum creatinine was 1.15 mg/dL on admission. The serum protein fractions showed an M peak at a high level and serum IgM was 1657 mg/dL. Protein electrophoresis showed IgM κ type M proteinemia. Although his monoclonal IgM was increased, bone marrow biopsy showed normal findings, with no infiltration of abnormal lymphocytes, in plasma cells, or chromosomal abnormalities, suggesting that this was a case of IgM monoclonal gammopathy rather than primary macroglobulinemia.

Because the patient did not agree to our proposal initially, a renal biopsy was performed 3 months after his first admission to elucidate the cause of his nephrotic syndrome. Thus, the etiology of his nephrosis was initially unknown due to the lack of renal biopsy. Considering his background, the possibility of membranous nephropathy was high; consequently, we administered 30 mg of oral prednisolone first. Three months after his first admission, the patient agreed to receive a renal biopsy.

**Fig. 1:**
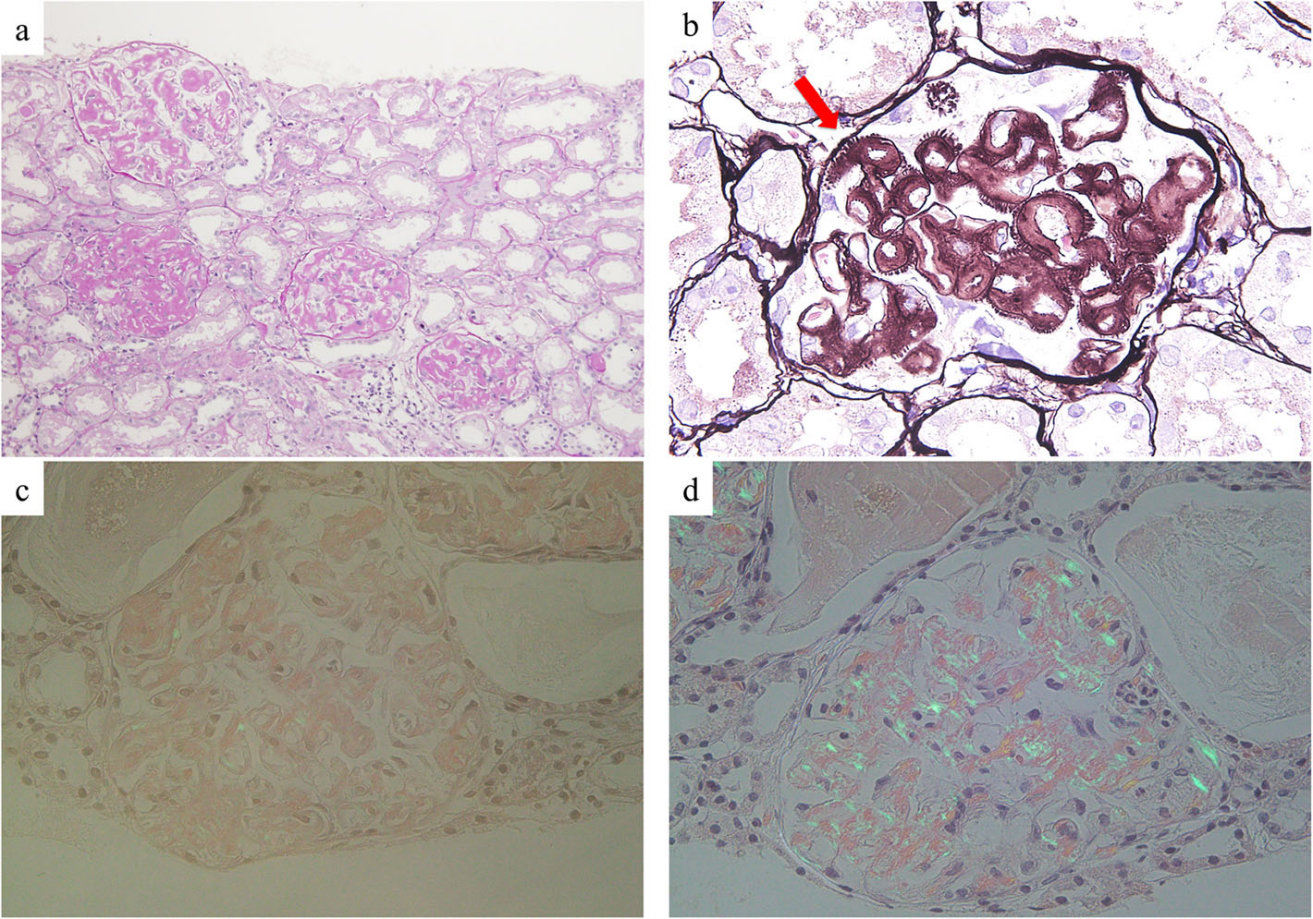
The gross pathological findings. (a) Periodic acid-Schiff staining, (b) Periodic acid-methenamine-silver staining, and polarized light microscope for (c) Direct Fast Scarlet staining and (**d**) Congo red staining

**Fig. 2:**
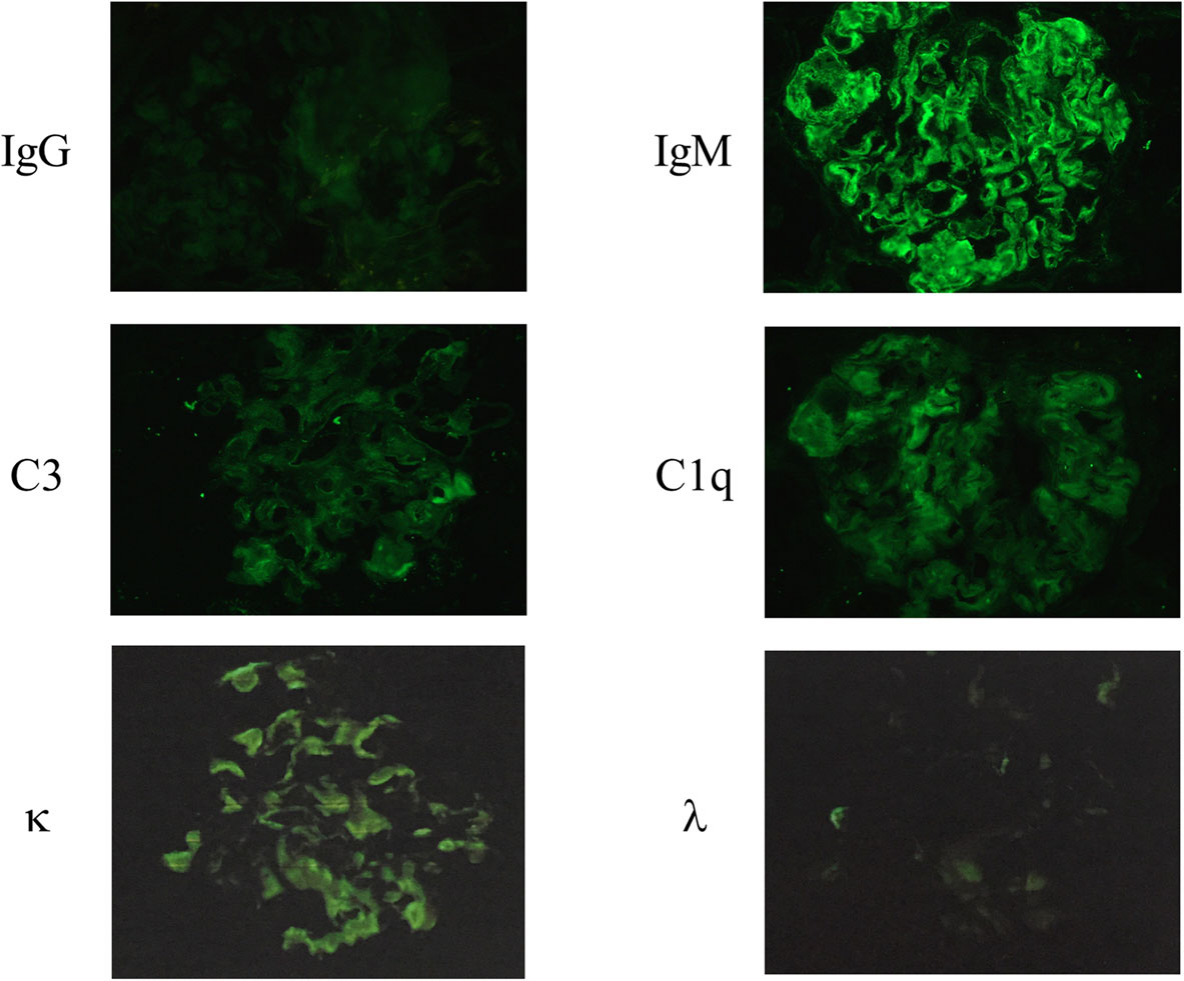
Immunostaining for IgG, IgM, C3, C1q, κ and λ. IgM and κ were positive in the mesangial and capillary loop

Under light microscopy, the biopsy sample largely consisted of renal cortex and contained 34 glomeruli. Marked enlargement of the mesangial region and strong periodic acid-Schiff (PAS)-positive deposits were observed on the glomerular basement membrane and mesangial matrix (Fig. 1a). Periodic acid-methenamine-silver (PAM) staining lead to strong silver staining of the spicula in the glomerular basement membrane and mesangial matrix area (Fig. 1b), but only weak DFS staining and scarce positive blue-green birefringence under polarized light microscope was observed (Fig. 1c). The immunofluorescence analysis showed that IgM and κ chains were clearly deposited on the glomerulus, and mild deposits were found in the mesangial region. No significant deposition of other globulins was observed; we considered this to be a case of monoclonal deposition of IgM-κ chain (Fig. 2).

**Fig. 3:**
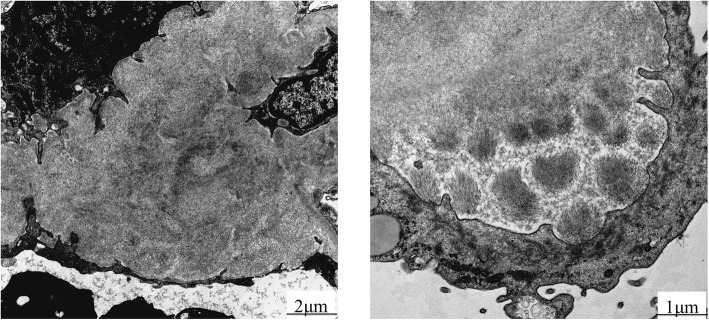
Electron microscopy findings. The arrow in the left panel shows the dense and scattered aggregates of amyloid fibrils in the subepithelial area. The right panel shows randomly oriented straight fibrils under high magnification

Electron microscopy showed deposition of fibrillar materials in the subepithelial and subepithelial regions of the mesangial and glomerular basement membranes. Deposition of fibrillar substances approximately 20 nm in diameter was also observed in the subendothelial and subepithelial layers of the mesangial and glomerular basement membrane by electron microscopy (Fig. 3). Based on the results of DFS staining and electron microscopy, the patient was diagnosed with FGN. This diagnosis was confirmed 6 months after his first admission because renal biopsy could not be performed at first admission. Further, it took approximately 2 months to receive the electron microscopy result. Since urinary protein did not improve after administration of oral prednisolone only, 150 mg of mizoribine was added as a drug for nephrosis. Temporary urinary protein decreased to approximately 4 g/gCr, but a significant increase in serum creatinine subsequently occurred. A daily dose of 100 mg of oral cyclophosphamide was started on the 180th day to halt disease progression of FGN and suppress the production of monoclonal IgM, resulting in a reduction of the increase in serum creatinine and a decrease in urinary protein. One month after initiation, the daily dose of cyclophosphamide was decreased to 50 mg and continued for 2 weeks. Soon after, the patient decided to cease administration of oral cyclophosphamide and oral prednisolone; the cumulative dose of cyclophosphamide was approximately 3.7 g. Although cyclophosphamide might have had a positive effect on renal prognosis, the progression of renal dysfunction could not be halted, and hemodialysis was initiated on the 230th day. The patient had melena caused by intestinal bleeding, the etiology of which was amyloidosis, but a diagnosis of AHL was not confirmed in the intestinal tissue. There was no symptom of nerve conduction from amyloidosis. The melena was not observed at the time of hemodialysis initiation, but occurred 2 years after hemodialysis initiation. In addition, the patient contracted liver cirrhosis and was troubled by ascites. Due to the severe intestinal bleeding and liver cirrhosis, his blood pressure decreased and finally he could not receive hemodialysis. The patient died from multiple organ failure at another hospital approximately 2 years after hemodialysis initiation. A cardiac echocardiogram performed 1 month before his death did not show evidence of cardiac amyloidosis, such as shaggy heart and cardiac wall thickening. Therefore, multiple organ failure was presumed to be mainly caused by the liver dysfunction.

**Fig. 4:**
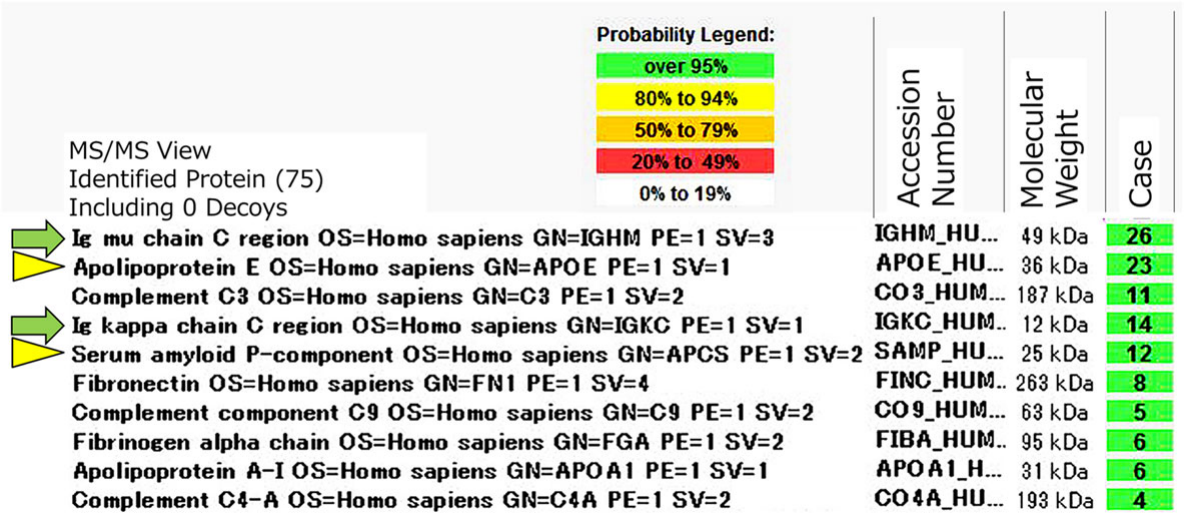
Result of LMD/MS according to the Scaffold database. Amyloid proteins (single μ heavy-chain and single κ light-chain: green colored arrows) were detected in conjunction with the amlyloidogenic proteins (serum amyloid P and apolipoprotein E: yellow colored arrowheads). LMD/MS: Laser microdissection followed by mass spectrometry

Based on the several new insights into FGN recently reported, we decided to reassess the renal biopsy. Even though DFS staining was weak positive, Congo red staining was positive, leading us to believe that this was a case of congophilic FGN. To confirm the diagnosis, the renal tissue was evaluated by LMD/MS and the Scaffold database, and a minimum of 4 mass spectra as described previously [9]. Notably, the presence of massive monoclonal heavy chain components (constant region), light chain components (constant region), serum amyloid P components, and apolipoprotein E (Apo E) components indicated that this was a case of heavy-and-light-chain amyloidosis (Fig. 4). No DNAJB9 component was detected. Although a blue-green birefringence under polarized light microscope in DFS staining was scarce (Fig. 1c), that in Congo red staining was positive (Fig. 1d).

## Discussion and conclusion

Here, we report a case with IgM monoclonal gammopathy who developed nephrotic syndrome. We initially diagnosed the patient with FGN; however, light microscopic examination of the renal biopsy showed PAM-positive amyloidosis, and a final diagnosis of AHL was confirmed by LMD/MS. Distinguishing FGN from amyloidosis is important because their causes, treatment, and prognosis are different [4]. In addition, determining the etiology of amyloid protein formation as well as accurate diagnosis and classification of the protein are crucial in amyloidosis [1].

LMD/MS has been widely recognized as a good approach to analyzing glomerular deposition disease [7, 10, 11]. The development of LMD/MS allows diagnosis of FGN even when Congo red staining is positive; without either LMD/MS or DNAJB9 staining, Congo red-positive FGN would be misdiagnosed as immunoglobulin light-chain amyloidosis (AL) [6].

Congo red staining was first used by Bennhold in 1922, and has since undergone several modifications to become established as the most commonly used approach for amyloid staining [8, 12]. It requires a positive control due to its relative instability and low sensitivity [12]. In contrast, DFS staining is known to be more sensitive and can detect small amounts of amyloid deposition, allowing its use as a substitute for Congo red staining [8]. We cannot speculate why a blue-green birefringence in DFS staining was scarcely stained in this case, but the presence of amyloid fibrils that were thicker than those observed in typical amyloid cases suggests that their structure may also be atypical.

The most common systemic amyloidosis is AL, which accounts for 85% of all cases of amyloidosis [13]. The second most common is reactive secondary amyloidosis (AA), which is caused by chronic inflammatory diseases. Immunoglobulin heavy-chain amyloidosis (AH) and AHL are rare.

The diagnosis of AHL should be limited only to cases that show strong equivalent staining for a single heavy chain and a single light chain under immunofluorescence [7]. It is sometime difficult for immunofluorescence to classify AHL, because the antibodies used in renal pathological examination tend to react to epitopes on the constant domain of immunoglobulins; if these epitopes have degenerated due to the presence of amyloid deposits, the staining will be negative. In addition, amyloid deposits may show weak nonspecific immunofluorescent staining for many immunoglobulin and complement components due to charge interactions and contamination [7].

There have been several case reports of AHL, but only 11 that were confirmed by LMD/MS. In these cases, massive amounts of heavy chain and light chain components were detected [7, 14, 15]. In this case, the heavy chain components were monoclonal and derived from the constant region, leading us to diagnose the patient with AHL. No previous case reports have described AHL-derived μ chain and κ chain confirmed by LMD/MS. Table 1 shows a summary of previously reported AHL cases that were confirmed by LMD/MS [7, 14, 15].

**Table 1:** Previously reported cases of renal heavy chain and light chain amyloidosis confirmed by LMD/MS

SE	Immunofluorescence	LMD/MS	reference
IgA, κ	IgA(2+), κ(2+)	IgA1 C + κ V + κ C	[7]
Neg	IgA(3+), IgG(1–2+), IgM(1+), C1q(1+), κ(1+)	IgA1 C+ κ C	[7]
IgA, κ	IgA(1+), C3(1+)	IgA1 C + κ V + κ C	[7]
IgA, λ	IgA(3+), λ(2+)	IgA1 C + λ V	[7]
λ	IgG(2–3+), λ(3+), κ(1+)	IgG1 C + λ V	[7]
IgG, λ	IgG(2+), C3(3+), λ(3+)	IgG1 C + λ V	[7]
IgG, λ	Neg	IgG1 C + λ V	[7]
IgG, λ	λ(3+)	IgG1 C + λ V	[7]
IgG, λ	IgG, C3, λ	IgG1 + λ	[14]
IgA, κ	IgA, κ	IgA + κ	[14]
IgG, λ	IgG, C3, λ	NA	[15]
IgM, κ	IgM, κ	IgM C + κ C	this case

*SE* Serum electrophoresis, *C* Constant domain, *V* Variable domain, *NA* Not available, *Neg* Negative, *LMD/MS*: Laser microdissection followed by mass spectrometry

Renal AHL is an uncommon and poorly recognized form of amyloidosis. Regarding the clinical features and prognosis of AHL and AL, Nasr et al. reported that AHL leads to fewer cardiac complications, responds better to treatment, and a longer survival time can be expected [16]. According to previous reports, the major etiologies of AHL and AH are plasma cell abnormalities [13]; however, in our patient the AHL amyloidosis was caused by IgM monoclonal gammopathy. The treatment for AHL is chemotherapy and stem cell transplantation, and hematologic response does not seem to differ from AL. In our case, lymphoproliferative disorder was not confirmed by bone marrow biopsy. Thus, a similar management to amyloidosis was not implemented and no cytotoxic drugs were administered. Although treatment with oral cyclophosphamide leads to a temporary reduction of urinary protein and slowing down of renal function decline, the serum creatinine did not improve. The patient ultimately progressed to end stage renal diseases and survived on hemodialysis for 2 years. Despite this case being initially misdiagnosed as FGN, we could not have selected the appropriate therapies because suitable treatments for FGN have not been confirmed and immunosuppressive therapies have only limited effect on renal prognosis [17, 18].

The diagnosis of this case was finally confirmed as AHL after the patient’s death; however, if this case had been diagnosed as monoclonal gammopathy of renal significance (MGRS), the patient’s prognosis could have been improved. MGRS includes various renal disorders characterized by the overproduction of monoclonal immunoglobulin; it does not always meet the criteria of B-cell proliferation, such as multiple myeloma, since the concept of MGRS was introduced to indicate monoclonal gammopathy with monoclonal immunoglobulins-associated renal disease in the absence of hematologic malignancy [19, 20]. Diagnosing MGRS allows earlier recognition of systematic disorders and improves patients’ prognosis via chemotherapy. The pathological findings of MGRS vary; renal lesions other than AL amyloidosis, such as light chain deposition disease, will also be included in MGRS [19, 20]. Since a precise diagnosis could not be performed in this case in a short period, cytotoxic treatment should have been considered from the view of MGRS. While LMD/MS is a powerful tool for precise diagnosis of amyloidosis, it cannot be used for all cases. Determining which cases would benefit from LMD/MS is based on several factors. First, if immunofluorescence findings are not clear, classification by LMD/MS is recommended [7]. Second, we should consider the availability of renal biopsy samples; LMD/MS is the only examination that can distinguish between AL, AH, AHL, AA, and FGN using paraffin blocks. Third, atypical findings may imply a rare type of amyloidosis. In this case, the amyloid deposits were PAM positive and the fibril diameter was thicker than that in typical AL cases. PAM-positive amyloidosis should be considered as a rare form of amyloidosis [13]. In such cases, LMD/MS would be useful for classification.

Although LMD/MS is a powerful modality for renal pathological examination, caution is required when interpreting its results. Manabe et al. reported a case of AL mimicking AHL, where a final diagnosis of AL could only be made after amyloid purification [21]. Amyloid purification was not performed in this case, but massive monoclonal heavy chain, light chain, serum amyloid P, and Apo E comments indicated that this was a case of AHL, as previously described [7].

In conclusion, we report here a case of AHL due to IgM monoclonal gammopathy that resembled FGN. Although the concept of MGRS may allow us to treat patients with overproduction of monoclonal immunoglobulin by chemotherapy, distinguishing amyloidosis from other diseases and classifying it precisely is crucial to determining the appropriate treatment strategy. In cases similar to ours, diagnosis may be difficult and LMD/MS can be a powerful tool for confirming the diagnosis.

## Data Availability

Anonymized data can be provided from the corresponding author on reasonable request.

## Abbreviations

AH: Immunoglobulin heavy-chain amyloidosis
AHL: Immunoglobulin heavy-and-light chain amyloidosis
AL: Immunoglobulin light-chain amyloidosis
DFS: Direct fast scarlet
FGN: Fibrillary glomerulonephritis
LMD/MS: Laser microdissection followed by mass spectrometry
MGRS: Monoclonal gammopathy of renal significance
PAS: Periodic acid-Schiff
PAM: Periodic acid-methenamine-silver
